# Supplementary material for: De novo adipocyte differentiation from Pdgfrβ+ preadipocytes protects against pathologic visceral adipose expansion in obesity
Source: Nat Commun. 2018 Mar 1;9:890. doi: 10.1038/s41467-018-03196-x (PMC5832777; doi:10.1038/s41467-018-03196-x)
Supplement: Supplementary file 1 — Description of Additional Supplementary Files [file 41467_2018_3196_MOESM1_ESM.docx]

**Description of Additional Supplementary Files**

File name: Supplementary Data 1.

Description: Summary of statistical data from this study.
