# Supplementary material for: De novo adipocyte differentiation from Pdgfrβ+ preadipocytes protects against pathologic visceral adipose expansion in obesity
Source: Nat Commun. 2018 Mar 1;9:890. doi: 10.1038/s41467-018-03196-x (PMC5832777; doi:10.1038/s41467-018-03196-x)
Supplement: Supplementary file 2 — Supplementary Information [file 41467_2018_3196_MOESM2_ESM.pdf]

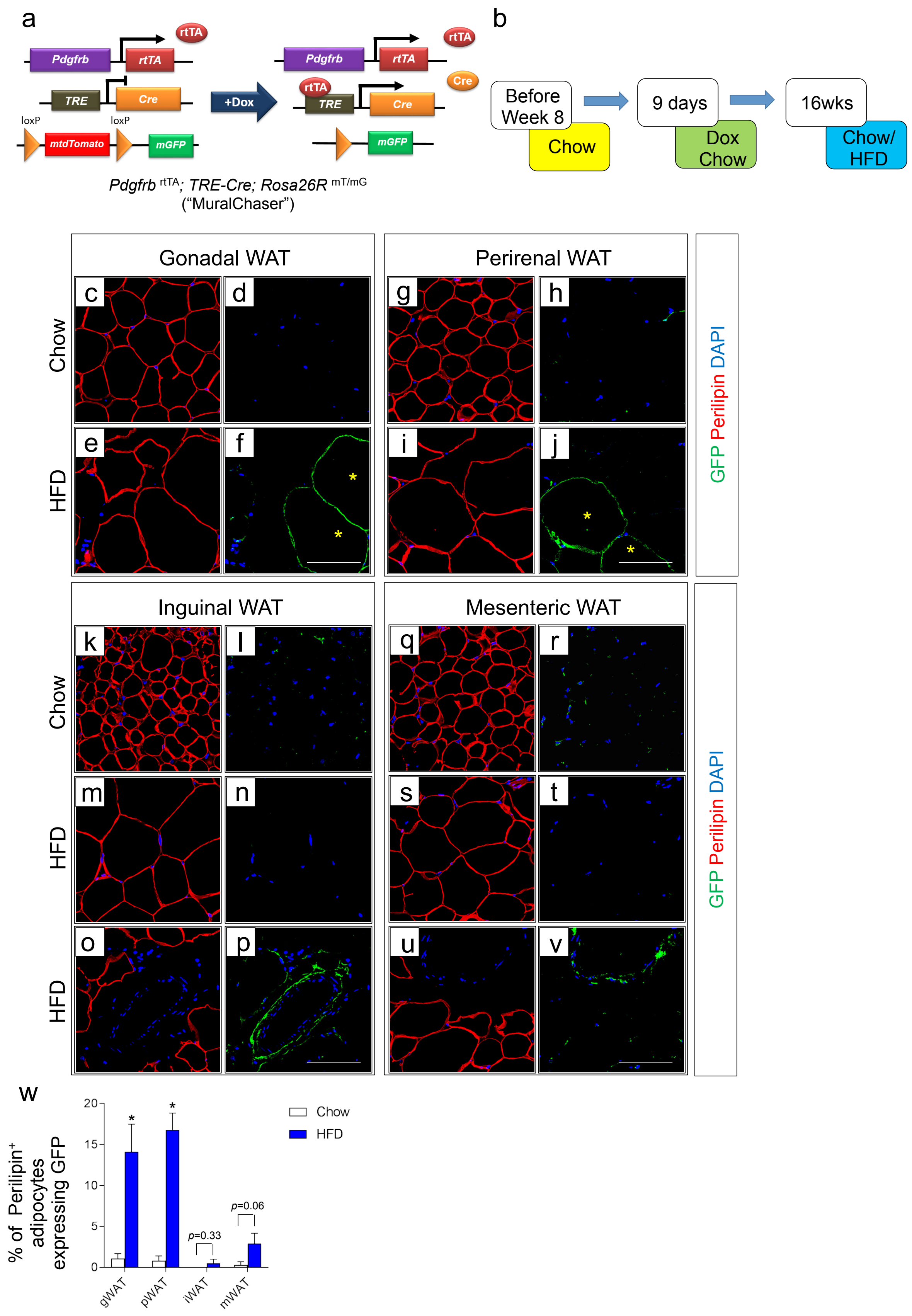
a
Pdgfrb
rtTA
TRE
Cre
loxP
mtdTomato
mGFP
+Dox
Pdgfrb rtTA; TRE-Cre; Rosa26R mT/mG
("MuralChaser")
b
Before Week 8
Chow
9 days
Dox Chow
16wks
Chow/ HFD
Gonadal WAT
Perirenal WAT
Inguinal WAT
Mesenteric WAT
Chow
HFD
GFP Perilipin DAPI
w
% of Perilipin+ adipocytes expressing GFP
Chow
HFD
p=0.33
p=0.06
gWAT
pWAT
iWAT
mWAT

**Supplementary Figure 1: Depot-selective contribution of Pdgfrβ+ precursors to visceral adipogenesis in obesity**

**(a)** The "MuralChaser" lineage tracing system is composed of *Pdgfrb* $^{rtTA}$, *TRE-Cre,* and *Rosa26R* $^{mT/mG}$ transgenic genes. In the presence of doxycycline (Dox), rtTA becomes transcriptionally active; active rtTA drives Cre expression and permanent activation of mGFP expression in Pdgfrβ$^{+}$ cells and descending cells.

**(b)** "Pulse-chase" lineage tracing approach. Muralchaser mice were fed on a standard chow diet until 8 weeks of age before being switched to dox-containing chow diet (600 mg/kg) for 9 days ("pulse"). Mice were then maintained on regular chow or HFD (devoid of Dox) for another 16 weeks ("chase") before adipose depots were harvested for paraffin sectioning. Processed paraffin sections were stained with anti-GFP (green) and anti-Perilipin (red) antibodies and counterstained with DAPI (blue; nuclei).

**(c-f)** Representative 63x magnification confocal immunofluorescence images of gonadal WAT sections after the "chase" period. * indicates GFP-labeled perilipin-positive cells. Scale bar, 50 μm.

**(g-j)** Representative 63x magnification confocal immunofluorescence images of perirenal WAT sections after the "chase" period. * indicates GFP-labeled perilipin-positive cells. Scale bar, 50 μm.

**(k-p)** Representative 63x magnification confocal immunofluorescence images of inguinal WAT sections after the "chase" period. Scale bar, 50 μm.

**(q-v)** Representative 63x magnification confocal immunofluorescence images of mesenteric WAT sections after the "chase" period. Scale bar, 50 μm.

**(w)** Percentage of perilipin-positive adipocytes expressing GFP in the indicated fat depots. Welch's t-test, *$p< 0.05$; n=12 randomly chosen 60x magnification fields from 2 individual reporter animals. Bars represent mean ± s.e.m.

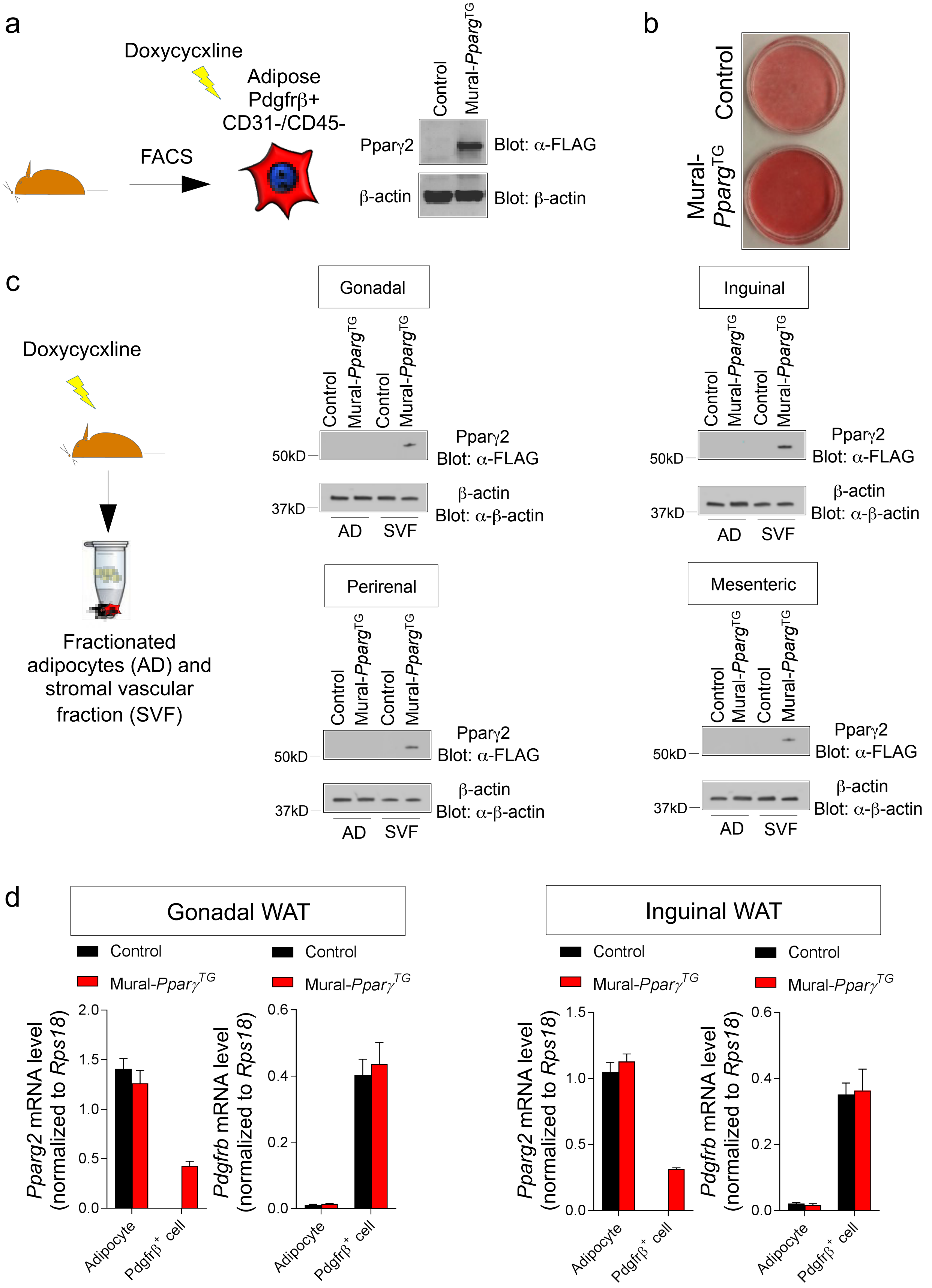

a
Doxycycxline
Adipose Pdgfrβ+ CD31-/CD45-
FACS
Control
Mural-Pparg^TG
Pparγ2
Blot: α-FLAG
β-actin
Blot: β-actin
b
Control
Mural-Pparg^TG
c
Doxycycxline
Fractionated adipocytes (AD) and stromal vascular fraction (SVF)
Gonadal
Inguinal
Perirenal
Mesenteric
Control
Mural-Pparg^TG
50kD
37kD
Pparγ2
Blot: α-FLAG
β-actin
Blot: α-β-actin
AD
SVF
d
Gonadal WAT
Inguinal WAT
Control
Mural-Pparγ^TG
Pparg2 mRNA level (normalized to Rps18)
Pdgfrb mRNA level (normalized to Rps18)
Adipocyte
Pdgfrβ+ cell

**Supplementary Figure 2: Doxycyline-inducible overexpression of *Pparg* in mural cells.**

**(a)** Pdgfrβ$^+$; CD45$^-$; CD31$^-$ cells were isolated from iWAT of 8 weeks-old control and Mural-*Pparg*$^{TG}$ mice fed on standard chow diet. Following the exposure of cells to doxycycline (10 μg/ml) for 48 hours *in vitro*, FLAG-tagged Pparγ2 protein expression in the cultured cells was determined by immunoblotting.

**(b)** Oil red staining of adipocytes (differentiation day 5) differentiated from iWAT Pdgfrβ$^+$; CD45$^-$; CD31$^-$ cells isolated and treated as described in **(a)**.

**(c)** Stromal vascular fraction (SVF) and adipocytes (AD) were fractionated from the indicated WAT depots of 8 weeks-old control and Mural-*Pparg*$^{TG}$ mice after 7 days of chow diet feeding containing doxycycline. FLAG-tagged Pparγ2 protein levels were determined by immunoblotting.

**(d)** mRNA levels of *Pparg2* and *Pdgfrb* in fractionated adipocytes and Pdgfrβ$^+$; CD45$^-$; CD31$^-$ cells isolated from gonadal and inguinal WAT of control and Mural-*Pparg*$^{TG}$ animals after 7 days of chow diet feeding containing doxycycline. * denotes $p<0.05$ from Welch's t-test. n=3 per genotype. Bars represent mean $\pm$ s.e.m.

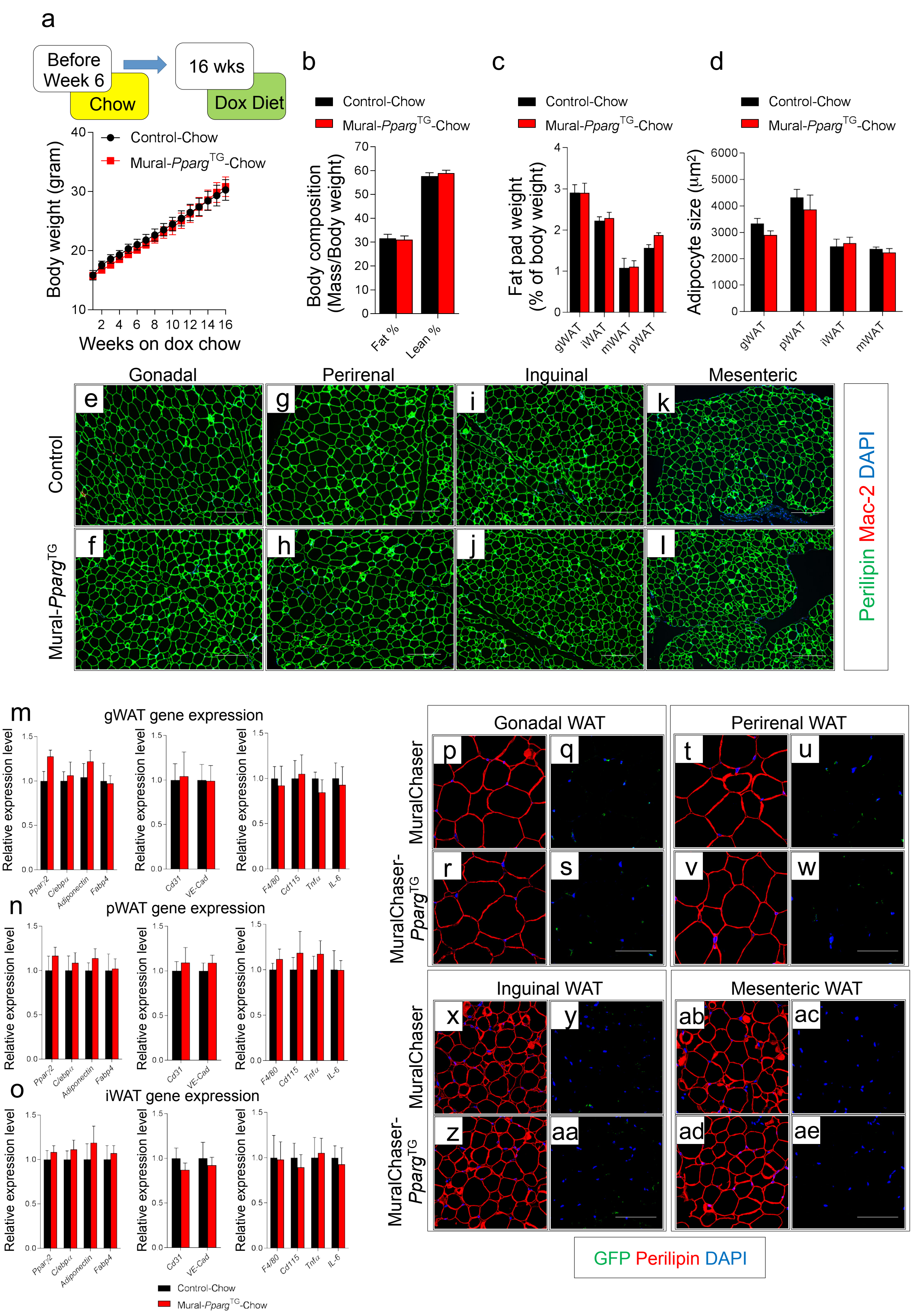
a
Before Week 6
Chow
16 wks
Dox Diet
Control-Chow
Mural-Pparg TG-Chow
Body weight (gram)
Weeks on dox chow
b
Body composition (Mass/Body weight)
Fat %
Lean %
c
Fat pad weight (% of body weight)
gWAT
iWAT
mWAT
pWAT
d
Adipocyte size (μm2)
Gonadal
Perirenal
Inguinal
Mesenteric
Control
Mural-Pparg TG
e
f
g
h
i
j
k
l
Perilipin Mac-2 DAPI
m
gWAT gene expression
n
pWAT gene expression
o
iWAT gene expression
Relative expression level
Pparγ2
C/ebpα
Adiponectin
Fabp4
Cd31
VE-Cad
F4/80
Cd115
Tnfα
IL-6
Control-Chow
Mural-Pparg TG-Chow
Gonadal WAT
Perirenal WAT
Inguinal WAT
Mesenteric WAT
MuralChaser
MuralChaser-Pparg TG
p
q
r
s
t
u
v
w
x
y
z
aa
ab
ac
ad
ae
GFP Perilipin DAPI

**Supplementary Figure 3: Mural *Pparg* overexpression does not drive adipocyte hyperplasia in lean mice**

**(a)** Control and Mural-*Pparg*$^{TG}$ mice were fed a standard chow diet until 6 weeks of age before being switched to dox-containing chow diet. Body weights were measured weekly following the onset of doxycycline treatment. n=7 per genotype. Data points represent mean $\pm$ s.e.m.

**(b)** Fat mass and lean mass (normalized to body weight) of control and Mural-*Pparg*$^{TG}$ mice after 16 weeks of dox-chow feeding. n=7 per genotype. Bars represent mean $\pm$ s.e.m.

**(c)** Fat pad weight (normalized to body weight) of control and Mural-*Pparg*$^{TG}$ mice after 16 weeks of dox-chow feeding. n=7 per genotype. Bars represent mean $\pm$ s.e.m.

**(d)** Average adipocyte size in indicated fat depots from control and Mural-*Pparg*$^{TG}$ mice after 16 weeks of dox-chow feeding. n=6 per genotype. Bars represent mean $\pm$ s.e.m.

**(e-l)** Representative immunofluorescence staining of Perilipin (green) and Mac-2 (red) in **(e-f)** gonadal, **(g-h)** perirenal, **(i-j)** inguinal and **(k-l)** mesenteric WAT paraffin sections obtained from control and Mural-*Pparg*$^{TG}$ mice after 16 weeks of dox-chow feeding. Scale bar, 200 μm.

**(m-o)** Relative mRNA levels of indicted genes in **(m)** gonadal, **(n)** perirenal and **(o)** inguinal WAT obtained from control and Mural-*Pparg*$^{TG}$ mice after 16 weeks of dox-chow diet feeding. n=6 per genotype. Bars represent mean $\pm$ s.e.m.

**(p-ae)** Control (MuralChaser) or MuralChaser-*Pparg*$^{TG}$ mice were kept on standard chow diet until 6 weeks of age before being switched to dox-containing chow diet for another 16 weeks. Fat depots were then harvested for paraffin sectioning. Processed paraffin sections were stained with anti-GFP (green) and anti-perilipin (red) and counterstained with DAPI (blue; nuclei). Representative 63x magnification confocal immunofluorescence images of **(p-s)** gonadal, **(t-w)** perirenal, **(x-aa)** inguinal and **(ab-ae)** mesenteric WAT sections after 16 weeks of dox-chow feeding. Scale bar, 50 μm.

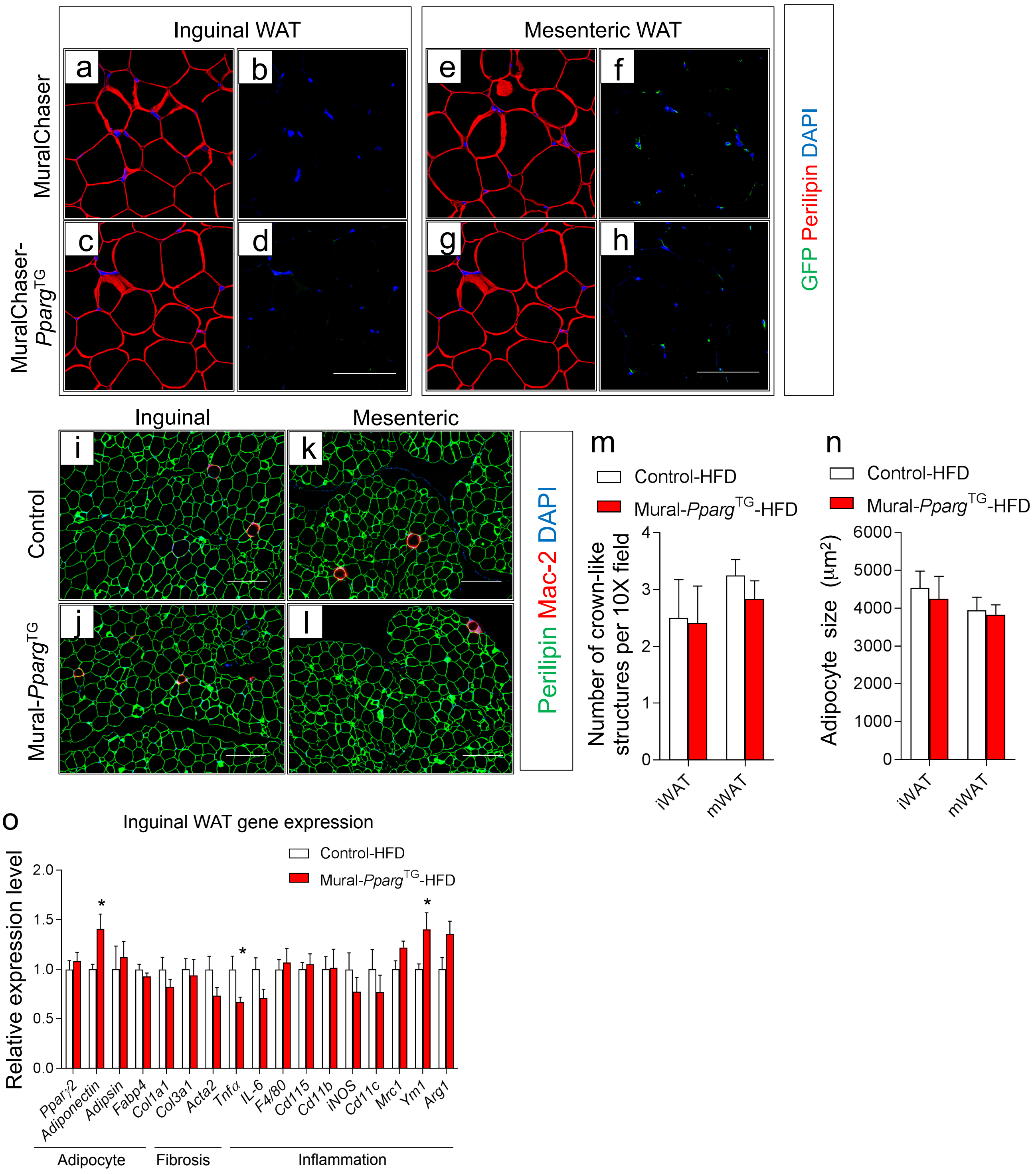
Inguinal WAT
Mesenteric WAT
MuralChaser
MuralChaser-PpargTG
a
b
c
d
e
f
g
h
GFP Perilipin DAPI
Inguinal
Mesenteric
Control
Mural-PpargTG
i
j
k
l
Perilipin Mac-2 DAPI
m
Control-HFD
Mural-PpargTG-HFD
Number of crown-like structures per 10X field
iWAT
mWAT
n
Adipocyte size (μm2)
o
Inguinal WAT gene expression
Relative expression level
Pparγ2
Adiponectin
Adipsin
Fabp4
Col1a1
Col3a1
Acta2
Tnfα
IL-6
F4/80
Cd115
Cd11b
iNOS
Cd11c
Mrc1
Ym1
Arg1
Adipocyte
Fibrosis
Inflammation

**Supplementary Figure 4: Inguinal and mesenteric WAT depots from obese Mural-*Pparg*$^{TG}$ mice**

**(a-h)** Representative 63x magnification confocal immunofluorescence images of **(a-d)** inguinal and **(e-h)** mesenteric WAT sections from MuralChaser-*Pparg*$^{TG}$ mice after 16 weeks dox-diet feeding. Sections were stained with anti-GFP (green) and anti-Perilipin (red) antibodies and counterstained with DAPI (blue; nuclei). Scale bar, 50 μm.

**(i-l)** Representative immunofluorescence staining of Perilipin (green) and Mac-2 (red) in **(i-j)** inguinal and **(k-l)** mesenteric WAT paraffin sections obtained from control and Mural-*Pparg*$^{TG}$ mice after 16 weeks of dox-HFD feeding. Scale bar, 200 μm.

**(m)** Number of crown-like structures (Mac-2 positive) in the indicated depots from control and Mural-*Pparg*$^{TG}$ mice after 16 weeks of dox-HFD feeding. * denotes $p<0.05$ from Welch's t-test. n=24 randomly chosen 10x magnification fields from 6 individual animals. Bars represent mean $\pm$ s.e.m.

**(n)** Average adipocyte size in indicated fat depots from control and Mural-*Pparg*$^{TG}$ mice after 16 weeks of dox-diet feeding. Control-HFD, n=8; Mural-*Pparg*$^{TG}$ -HFD, n=9. Bars represent mean $\pm$ s.e.m.

**(o)** Relative mRNA levels of indicted genes in inguinal WAT obtained from control and Mural-*Pparg*$^{TG}$ mice after 16 weeks of dox-HFD feeding. * denotes $p<0.05$ from Welch's t-test. n=6 per genotype. Bars represent mean $\pm$ s.e.m.

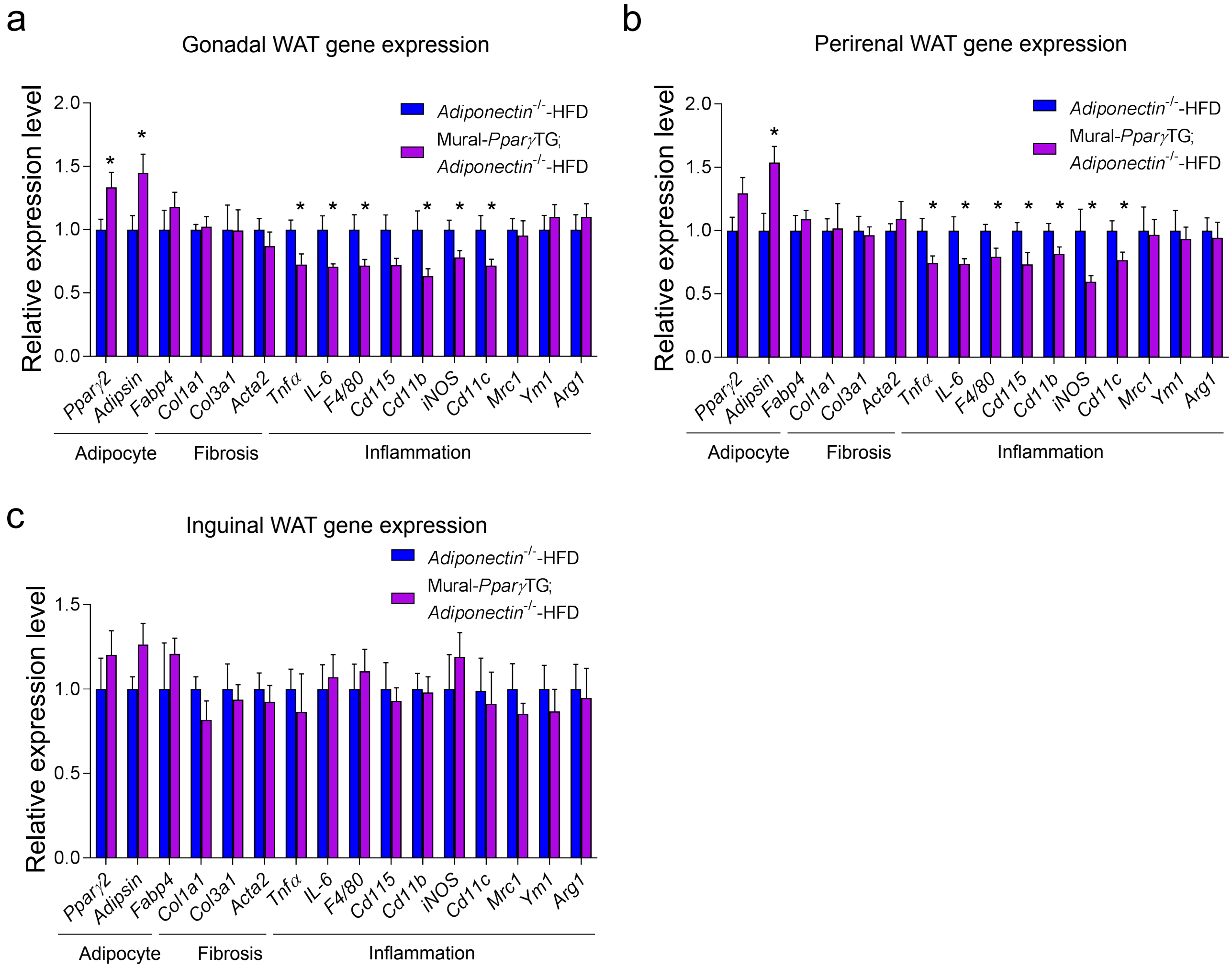


**Supplementary Figure 5: Adipose gene expression in obese *Adiponectin*$^{-/-}$ and *Adiponectin*$^{-/-}$; Mural-*Pparγ*$^{TG}$ mice.**

**(a)** Relative mRNA levels of indicted genes in gonadal WAT obtained from *Adiponectin*$^{-/-}$ and *Adiponectin*$^{-/-}$; Mural-*Pparg*$^{TG}$ mice after 16 weeks of dox-HFD feeding. * denotes $p<0.05$ from Welch's t-test. n=6 per genotype. Bars represent mean $\pm$ s.e.m.

**(b)** Relative mRNA levels of indicted genes in perirenal WAT obtained from *Adiponectin*$^{-/-}$ and *Adiponectin*$^{-/-}$; Mural-*Pparg*$^{TG}$ mice after 16 weeks of dox-HFD feeding. * denotes $p<0.05$ from Welch's t-test. n=6 per genotype. Bars represent mean $\pm$ s.e.m.

**(c)** Relative mRNA levels of indicted genes in inguinal WAT obtained from *Adiponectin*$^{-/-}$ and *Adiponectin*$^{-/-}$; Mural-*Pparg*$^{TG}$ mice after 16 weeks of dox-HFD feeding. n=6 per genotype. Bars represent mean $\pm$ s.e.m.

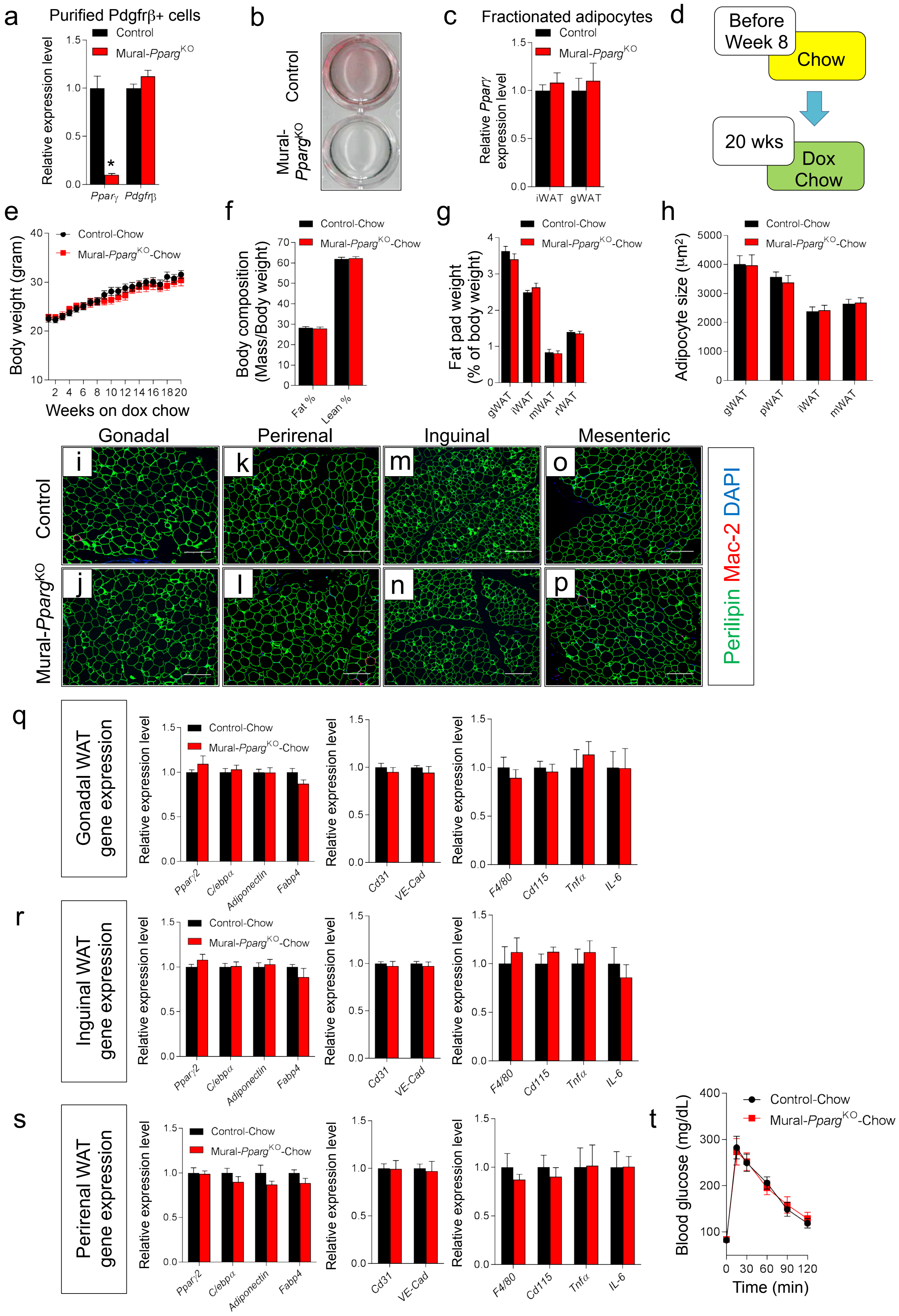

a
Purified Pdgfrβ+ cells
Control
Mural-Pparg KO
Relative expression level
Pparγ
Pdgfrβ
b
Control
Mural-Pparg KO
c
Fractionated adipocytes
Control
Mural-Pparg KO
Relative Pparγ expression level
iWAT
gWAT
d
Before Week 8
Chow
20 wks
Dox Chow
e
Control-Chow
Mural-Pparg KO-Chow
Body weight (gram)
Weeks on dox chow
f
Control-Chow
Mural-Pparg KO-Chow
Body composition (Mass/Body weight)
Fat %
Lean %
g
Control-Chow
Mural-Pparg KO-Chow
Fat pad weight (% of body weight)
gWAT
iWAT
mWAT
rWAT
h
Control-Chow
Mural-Pparg KO-Chow
Adipocyte size (μm²)
gWAT
pWAT
iWAT
mWAT
Gonadal
Perirenal
Inguinal
Mesenteric
Control
Mural-Pparg KO
Perilipin Mac-2 DAPI
q
Gonadal WAT gene expression
r
Inguinal WAT gene expression
s
Perirenal WAT gene expression
Control-Chow
Mural-Pparg KO-Chow
Relative expression level
Pparγ2
C/ebpα
Adiponectin
Fabp4
Cd31
VE-Cad
F4/80
Cd115
Tnfα
IL-6
t
Control-Chow
Mural-Pparg KO-Chow
Blood glucose (mg/dL)
Time (min)

**Supplementary Figure 6: Mural *Pparg* is dispensable for WAT homeostasis in lean adult mice.**

**(a)** Relative mRNA levels of *Pparg* and *Pdgfrb* in Pdgfrβ$^+$; Lin$^-$ (CD31$^-$/CD45$^-$) cells isolated from iWAT of 8 weeks-old control and Mural-*Pparg*$^{KO}$ mice after 7 days of dox-chow feeding. * denotes p<0.05 from Welch's t-test. n=3 per genotype. Bars represent mean ± s.e.m.

**(b)** Oil red staining of adipocytes (differentiation day 7) differentiated from iWAT Pdgfrβ$^+$; Lin$^-$ cells isolated from 8 weeks-old control and Mural-*Pparg*$^{KO}$ mice after 7 days of dox-chow feeding.

**(c)** Relative mRNA levels of *Pparg* in fractionated adipocytes isolated from iWAT or gWAT of 8 weeks-old control and Mural-*Pparg*$^{KO}$ mice after 7 days of dox-chow feeding. n=4 per genotype. Bars represent mean ± s.e.m.

**(d)** Control and Mural-*Pparg*$^{KO}$ animals were kept at room temperature and fed on standard normal chow until 8 weeks of age before being switched to dox-containing chow for another 20 weeks.

**(e)** Body weights of control and Mural-*Pparg*$^{KO}$ mice during 20 weeks of dox-chow feeding. Control-Chow, n=8; Mural-*Pparg*$^{KO}$ -Chow, n=7. Data points represent mean ± s.e.m.

**(f)** Fat mass and lean mass (normalized to body weight) of control and Mural-*Pparg*$^{KO}$ mice after 20 weeks of dox-chow feeding. Control-Chow, n=8; Mural-*Pparg*$^{KO}$ -Chow, n=7. Bars represent mean ± s.e.m.

**(g)** Fat pad weight (normalized to body weight) of control and Mural-*Pparg*$^{KO}$ mice after 20 weeks of dox-chow feeding. Control-Chow, n=8; Mural-*Pparg*$^{KO}$ -Chow, n=7. Bars represent mean ± s.e.m.

**(h)** Average adipocyte size in indicated fat depots from control and Mural-*Pparg*$^{KO}$ mice after 20 weeks of dox-chow feeding. n=6 per genotype. Bars represent mean ± s.e.m.

**(i-p)** Representative immunofluorescence staining of Perilipin (green) and Mac-2 (red) in **(i-j)** gonadal, **(k-l)** perirenal, **(m-n)** inguinal and **(o-p)** mesenteric WAT paraffin sections obtained from control and Mural-*Pparg*$^{KO}$ mice after 20 weeks of dox-chow diet feeding. Scale bar, 200 μm.

**(q-s)** Relative mRNA levels of indicted genes in **(m)** gonadal, **(n)** inguinal and **(o)** perirenal WAT obtained from control and Mural-*Pparg*$^{KO}$ mice after 20 weeks of dox-chow diet feeding. n=6 per genotype. Bars represent mean ± s.e.m.

**(t)** Glucose tolerance tests of control and Mural-*Pparg*$^{KO}$ mice after 20 weeks of dox-chow feeding. Control-Chow, n=8; Mural-*Pparg*$^{KO}$ -Chow, n=7. Data points represent mean ± s.e.m.

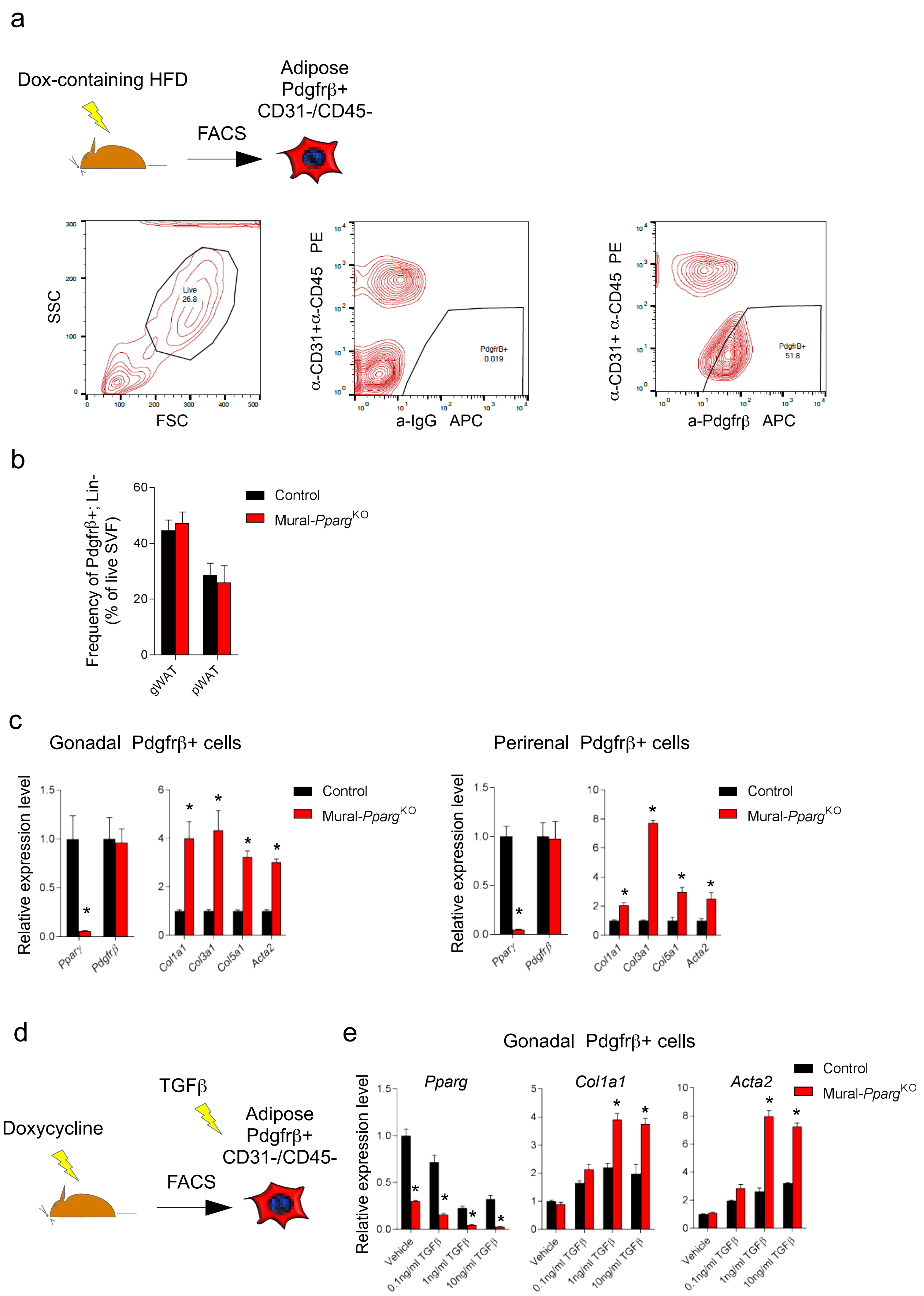

a
Dox-containing HFD
FACS
Adipose
Pdgfrβ+
CD31-/CD45-
SSC
FSC
Live
26.8
α-CD31+α-CD45 PE
a-IgG APC
PdgfrB+
0.019
α-CD31+ α-CD45 PE
a-Pdgfrβ APC
PdgfrB+
51.8
b
Frequency of Pdgfrβ+; Lin-
(% of live SVF)
Control
Mural-Pparg KO
gWAT
pWAT
c
Gonadal Pdgfrβ+ cells
Perirenal Pdgfrβ+ cells
Relative expression level
Control
Mural-Pparg KO
Pparγ
Pdgfrβ
Col1a1
Col3a1
Col5a1
Acta2
d
Doxycycline
TGFβ
FACS
Adipose
Pdgfrβ+
CD31-/CD45-
e
Gonadal Pdgfrβ+ cells
Pparg
Col1a1
Acta2
Relative expression level
Control
Mural-Pparg KO
Vehicle
0.1ng/ml TGFβ
1ng/ml TGFβ
10ng/ml TGFβ

**Supplementary Figure 7: *Pparg*–deficient Pdgfrβ+ cells adopt a myofibroblast-like phenotype.**

**(a)** Pdgfrβ$^{+}$; Lin$^{-}$ cells were isolated from fat depots of control and Mural-*Pparg*$^{KO}$ mice after 20 weeks of dox-HFD feeding. 100,000 Pdgfrβ$^{+}$; Lin$^{-}$ cells isolated from pooled stromal vascular cells of 2 individual fat depots were utilized for subsequent qPCR analysis and considered as one individual measurement. Representative FACS collection gates for the isolation of Pdgfrβ$^{+}$; Lin$^{-}$ cells are shown.

**(b)** Frequency of Pdgfrβ$^{+}$; Lin$^{-}$ cells among live SVF cells isolated from the indicated fat depots. n=3 per genotype. Bars represent mean $\pm$ s.e.m.

**(c)** Relative mRNA levels of *Pparg*, *Pdgfrb,* and the indicated fibrosis-related genes in Pdgfrβ$^{+}$; Lin$^{-}$ cells isolated from gonadal WAT and perirenal WAT of control and Mural-*Pparg*$^{KO}$ mice after 20 weeks of dox-HFD feeding. * denotes $p<0.05$ from Welch's t-test. n=3 per genotype. Bars represent mean $\pm$ s.e.m.

**(d)** Control and Mural-*Pparg*$^{KO}$ animals were kept at room temperature and fed on standard normal chow until 8 weeks of age before being switched to dox-containing chow for 7 days. Pdgfrβ$^{+}$; Lin$^{-}$ cells isolated from gonadal WAT of control and Mural-*Pparg*$^{KO}$ mice after dox-chow feeding were cultured and treated with TGFβ for 3 days.

**(e)** Relative mRNA levels of the indicated genes after TGFβ treatment of Pdgfrβ$^{+}$; Lin$^{-}$ cells isolated from gonadal WAT of control and Mural-*Pparg*$^{KO}$ mice. * denotes $p<0.05$ from Student's t-test. n=3 per genotype. Bars represent mean $\pm$ s.e.m.

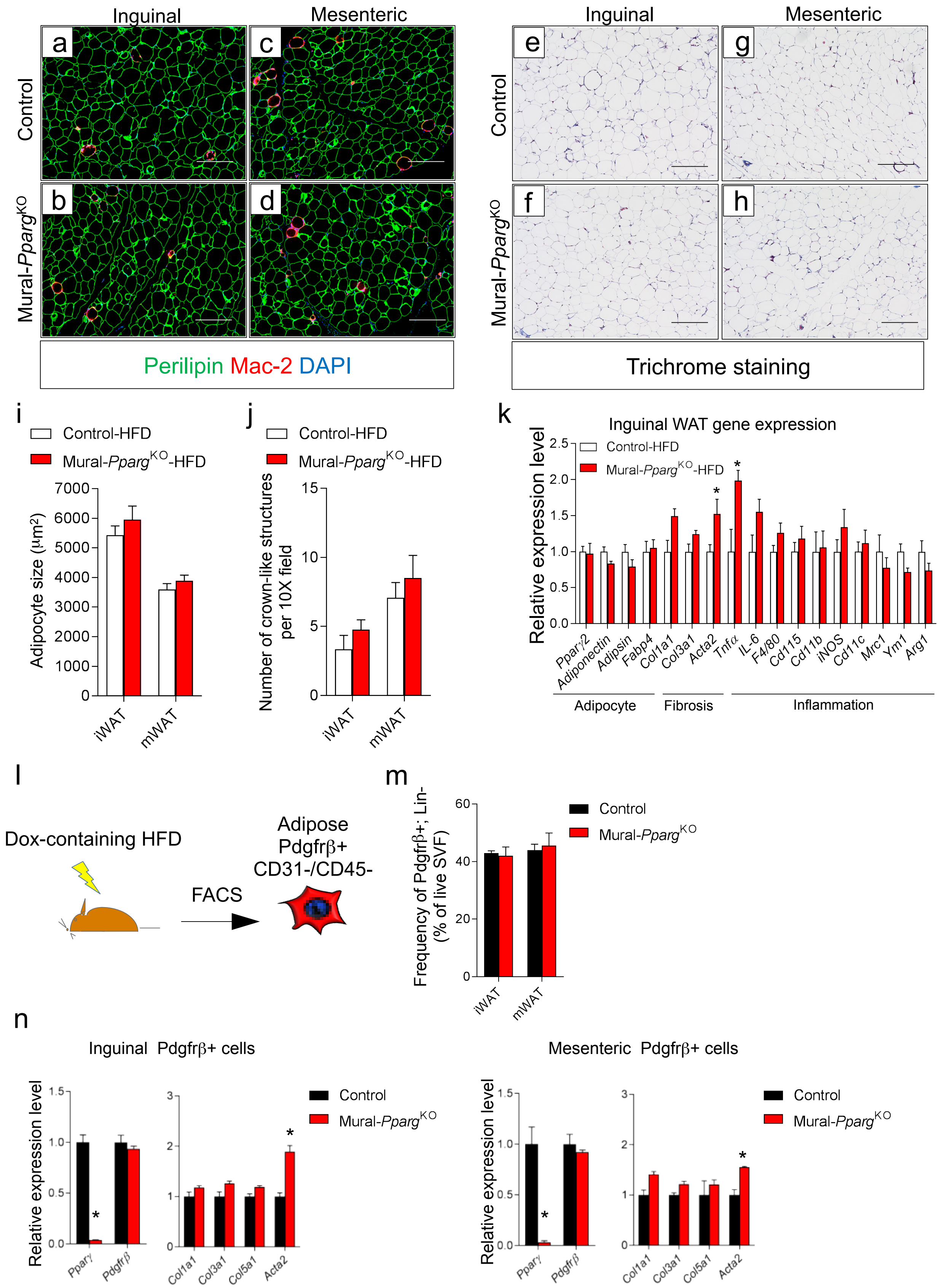

Inguinal
Mesenteric
Control
Mural-Pparg^KO
a
b
c
d
Perilipin Mac-2 DAPI
Inguinal
Mesenteric
Control
Mural-Pparg^KO
e
f
g
h
Trichrome staining
i
Control-HFD
Mural-Pparg^KO-HFD
Adipocyte size (µm²)
iWAT
mWAT
j
Control-HFD
Mural-Pparg^KO-HFD
Number of crown-like structures per 10X field
iWAT
mWAT
k
Inguinal WAT gene expression
Control-HFD
Mural-Pparg^KO-HFD
Relative expression level
Pparγ2
Adiponectin
Adipsin
Fabp4
Col1a1
Col3a1
Acta2
Tnfα
IL-6
F4/80
Cd115
Cd11b
iNOS
Cd11c
Mrc1
Ym1
Arg1
Adipocyte
Fibrosis
Inflammation
l
Dox-containing HFD
FACS
Adipose Pdgfrβ+ CD31-/CD45-
m
Frequency of Pdgfrβ+; Lin- (% of live SVF)
Control
Mural-Pparg^KO
iWAT
mWAT
n
Inguinal Pdgfrβ+ cells
Relative expression level
Pparγ
Pdgfrβ
Col1a1
Col3a1
Col5a1
Acta2
Control
Mural-Pparg^KO
Mesenteric Pdgfrβ+ cells
Relative expression level
Pparγ
Pdgfrβ
Col1a1
Col3a1
Col5a1
Acta2
Control
Mural-Pparg^KO

**Supplementary Figure 8: Inguinal and mesenteric WAT depots from obese Mural-*Pparg*$^{KO}$ mice**

**(a-d)** Representative immunofluorescence staining of Perilipin (green) and Mac-2 (red) in **(a-b)** inguinal and **(c-d)** mesenteric WAT paraffin sections obtained from control and Mural-*Pparg*$^{KO}$ mice after 20 weeks of dox-HFD feeding. Scale bar, 200 μm.

**(e-h)** Representative trichrome staining of **(e-f)** inguinal and **(g-h)** mesenteric WAT paraffin sections obtained from control and Mural-*Pparg*$^{KO}$ mice after 20 weeks of dox-HFD feeding. Scale bar, 200 μm.

**(i)** Number of crown-like structures (Mac-2 positive) in the indicated depots obtained from control and Mural-*Pparg*$^{KO}$ mice after 20 weeks of dox-HFD feeding. n=24 randomly chosen 10x magnification fields from 6 individual animals. Bars represent mean $\pm$ s.e.m.

**(j)** Average adipocyte size in indicated fat depots from control and Mural-*Pparg*$^{KO}$ mice after 20 weeks of dox-HFD feeding. Control-HFD, n=8; Mural-*Pparg*$^{KO}$ -HFD, n=7. Bars represent mean $\pm$ s.e.m.

**(k)** Relative mRNA levels of indicted genes in inguinal WAT obtained from control and Mural-*Pparg*$^{KO}$ mice after 20 weeks of dox-HFD diet feeding. * denotes $p<0.05$ from Welch's t-test. n=6 per genotype. Bars represent mean $\pm$ s.e.m.

**(l)** Pdgfr$\beta^{+}$; Lin$^{-}$ cells were isolated from inguinal and mesenteric fat depots of control and Mural-*Pparg*$^{KO}$ mice after 20 weeks of dox-HFD feeding and analyzed as described in Supplemental Figure 7a.

**(m)** Frequency of Pdgfr$\beta^{+}$; Lin$^{-}$ cells among live SVF cells isolated from the indicated fat depots. Bars represent mean $\pm$ s.e.m.

**(n)** Relative mRNA levels of *Pparg*, *Pdgfrb,* and the indicated fibrosis-related genes in Pdgfr$\beta^{+}$; Lin$^{-}$ cells isolated from inguinal WAT and mesenteric WAT of control and Mural-*Pparg*$^{KO}$ mice after 20 weeks of dox-HFD feeding. * denotes $p<0.05$ from Student's t-test. n=3 per genotype. Bars represent mean $\pm$ s.e.m.

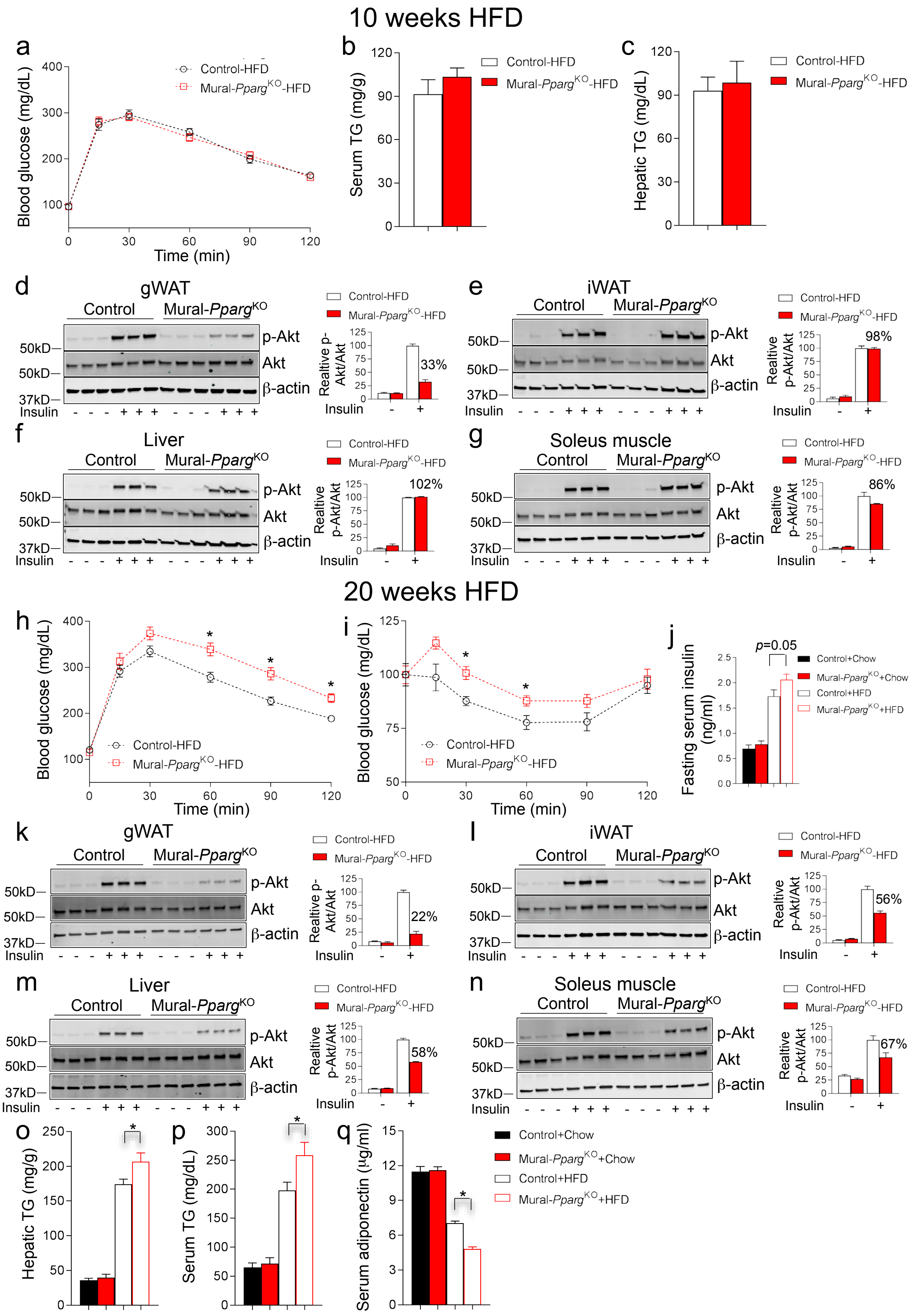

10 weeks HFD
a
Blood glucose (mg/dL)
Time (min)
Control-HFD
Mural-Pparg^KO-HFD
b
Serum TG (mg/g)
c
Hepatic TG (mg/dL)
d
gWAT
Control
Mural-Pparg^KO
p-Akt
Akt
β-actin
50kD
37kD
Insulin
Realtive p-Akt/Akt
33%
e
iWAT
98%
f
Liver
102%
g
Soleus muscle
86%
20 weeks HFD
h
i
j
Fasting serum insulin (ng/ml)
p=0.05
Control+Chow
Mural-Pparg^KO+Chow
Control+HFD
Mural-Pparg^KO+HFD
k
22%
l
56%
m
58%
n
67%
o
Hepatic TG (mg/g)
p
Serum TG (mg/dL)
q
Serum adiponectin (µg/ml)

**Supplementary Figure 9: Obese Mural-*Pparg*$^{KO}$ mice are glucose intolerant and insulin resistant.**

**(a)** Glucose tolerance tests of control and Mural-*Pparg*$^{KO}$ mice after 10 weeks of dox-HFD feeding. Control-HFD, n=14; Mural-*Pparg*$^{KO}$ -HFD, n=13. Data points represent mean ± s.e.m.

**(b)** Serum and **(c)** hepatic triglycerides levels in control and Mural-*Pparg*$^{KO}$ mice after 10 weeks of dox-HFD feeding. n=5 per genotype. Bars represent mean ± s.e.m.

**(d-g)** Western blot analysis of phosphorylated Akt (p-Akt), total Akt, and b-actin protein levels in tissue extracts of **(d)** gonadal WAT, **(e)** inguinal WAT, **(f)** liver and **(g)** soleus muscle, from control and Mural-*Pparg*$^{KO}$ mice after 10 weeks of dox-HFD feeding. For quantification, the intensity of p-Akt band is normalized to that of total Akt band. The mean values of pAkt/Akt intensity ratio of insulin-treated control mice samples were set as 100%. n=3 individual mice per genotype. Bars represent mean ± s.e.m.

**(h)** Glucose tolerance tests of control and Mural-*Pparg*$^{KO}$ mice after 20 weeks of dox-HFD feeding. * denotes p<0.05 from Student's t-test. Control-HFD, n=14; Mural-*Pparg*$^{KO}$ -HFD, n=13. Data points represent mean ± s.e.m.

**(i)** Insulin tolerance test of control and Mural-*Pparg*$^{KO}$ mice after 20 weeks of dox-HFD feeding. * denotes p<0.05 from Student's t-test. Control-HFD, n=14; Mural-*Pparg*$^{KO}$ -HFD, n=13. Data points represent mean ± s.e.m.

**(j)** 6-hour fasting serum insulin levels in control and Mural-*Pparg*$^{KO}$ mice after 20 weeks of dox-HFD feeding. Control-Chow, n=8; Mural-*Pparg*$^{KO}$ -Chow, n=7; Control-HFD, n=8; Mural-*Pparg*$^{KO}$ -HFD, n=7. Bars represent mean ± s.e.m.

**(k-n)** Western blot analysis of phosphorylated Akt (p-Akt), total Akt and b-actin protein levels in tissue extracts of **(k)** gonadal WAT, **(l)** inguinal WAT, **(m)** liver and **(n)** soleus muscle from control and Mural-*Pparg*$^{KO}$ mice after 20 weeks of dox-HFD feeding. For quantification, the intensity of p-Akt band is normalized to that of total Akt band. The mean values of pAkt/Akt intensity ratio of insulin-treated control mice samples were set as 100%. n=3 individual mice per genotype. Bars represent mean ± s.e.m.

**(o)** Hepatic and **(p)** serum triglycerides levels in control and Mural-*Pparg*$^{KO}$ mice after 20 weeks of dox-diet feeding. Two-way ANOVA, *p< 0.05; Control-Chow, n=8; Mural-*Pparg*$^{KO}$ -Chow, n=7; Control-HFD, n=8; Mural-*Pparg*$^{KO}$ -HFD, n=7. Bars represent mean ± s.e.m.

**(q)** Serum adiponectin levels in control and Mural-*Pparg*$^{KO}$ mice after 20 weeks of dox-diet feeding. Two-way ANOVA, *p< 0.05; Control-Chow, n=8; Mural-*Pparg*$^{KO}$ -Chow, n=7; Control-HFD, n=14; Mural-*Pparg*$^{KO}$ -HFD, n=13. Bars represent mean ± s.e.m.

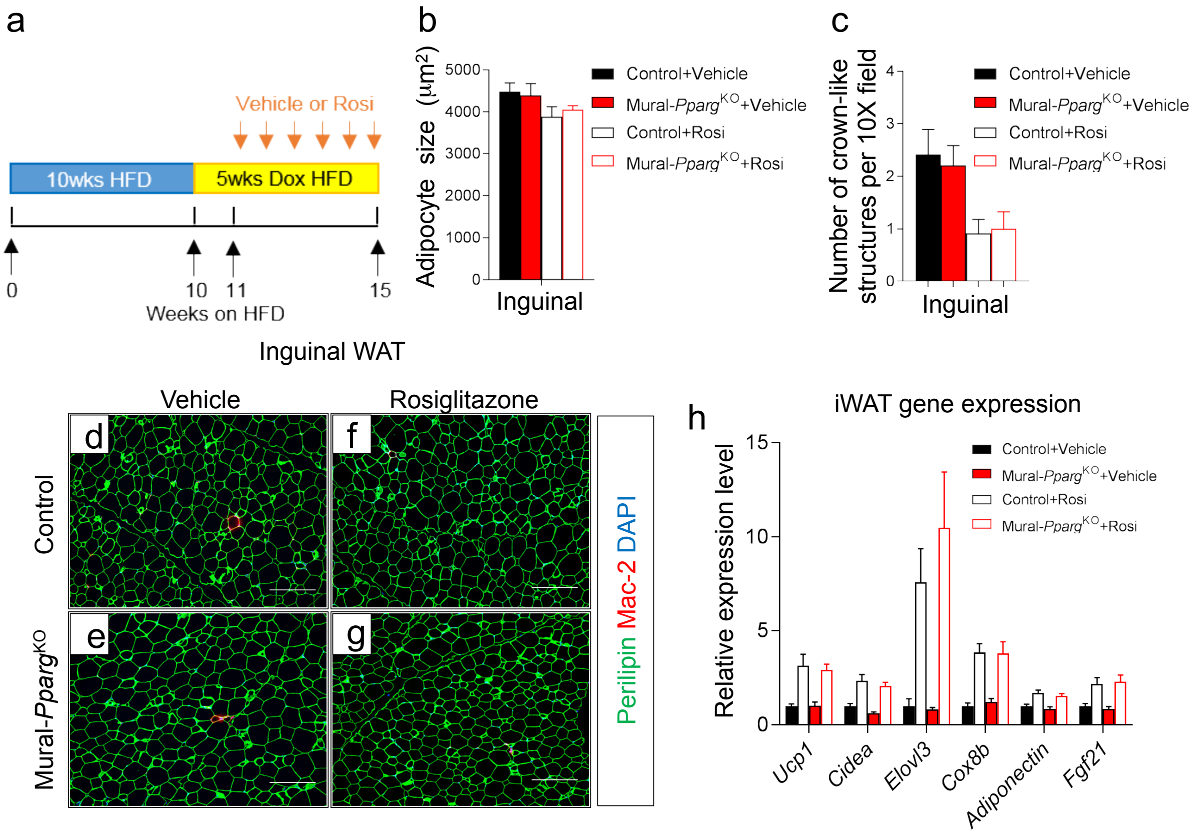


**Supplementary Figure 10: Analysis of inguinal WAT from obese TZD-treated Mural-*Pparg*$^{KO}$ mice**

**(a)** Control and Mural-*Pparg*$^{KO}$ mice were fed on standard chow diet until 6 weeks of age before being switched to HFD for 10 week. Mice were then fed dox-containing HFD for another 5 weeks. After 1 week of dox-HFD, vehicle (1% methylcellulose) or rosiglitazone (10mg/kg/day) were administrated daily to the animals for 4 weeks by oral gavage.

**(b)** Average adipocyte size in inguinal WAT from control and Mural-*Pparg*$^{KO}$ mice treated with vehicle or rosiglitazone. Control+Vehicle, n=6; Mural-*Pparg*$^{KO}$ +Vehicle, n=6; Control+Rosi, n=7; Mural-*Pparg*$^{KO}$ +Rosi, n=8. Bars represent mean $\pm$ s.e.m.

**(c)** Number of crown-like structures (Mac-2 positive) in inguinal WAT from control and Mural-*Pparg*$^{KO}$ mice treated with vehicle or rosiglitazone. n=24 randomly chosen 10x magnification fields from 6 individual animals. Bars represent mean $\pm$ s.e.m.

**(d-g)** Representative immunofluorescence staining of Perilipin (green) and Mac-2 (red) in inguinal WAT paraffin sections obtained from control and Mural-*Pparg*$^{KO}$ mice after 4 weeks of vehicle or rosiglitazone treatment. Scale bar, 200 µm.

**(h)** Relative mRNA levels of indicated rosiglitazone-induced genes in inguinal WAT obtained from control and Mural-*Pparg*$^{KO}$ mice treated with vehicle or rosiglitazone. n=6 per group. Bars represent mean $\pm$ s.e.m.

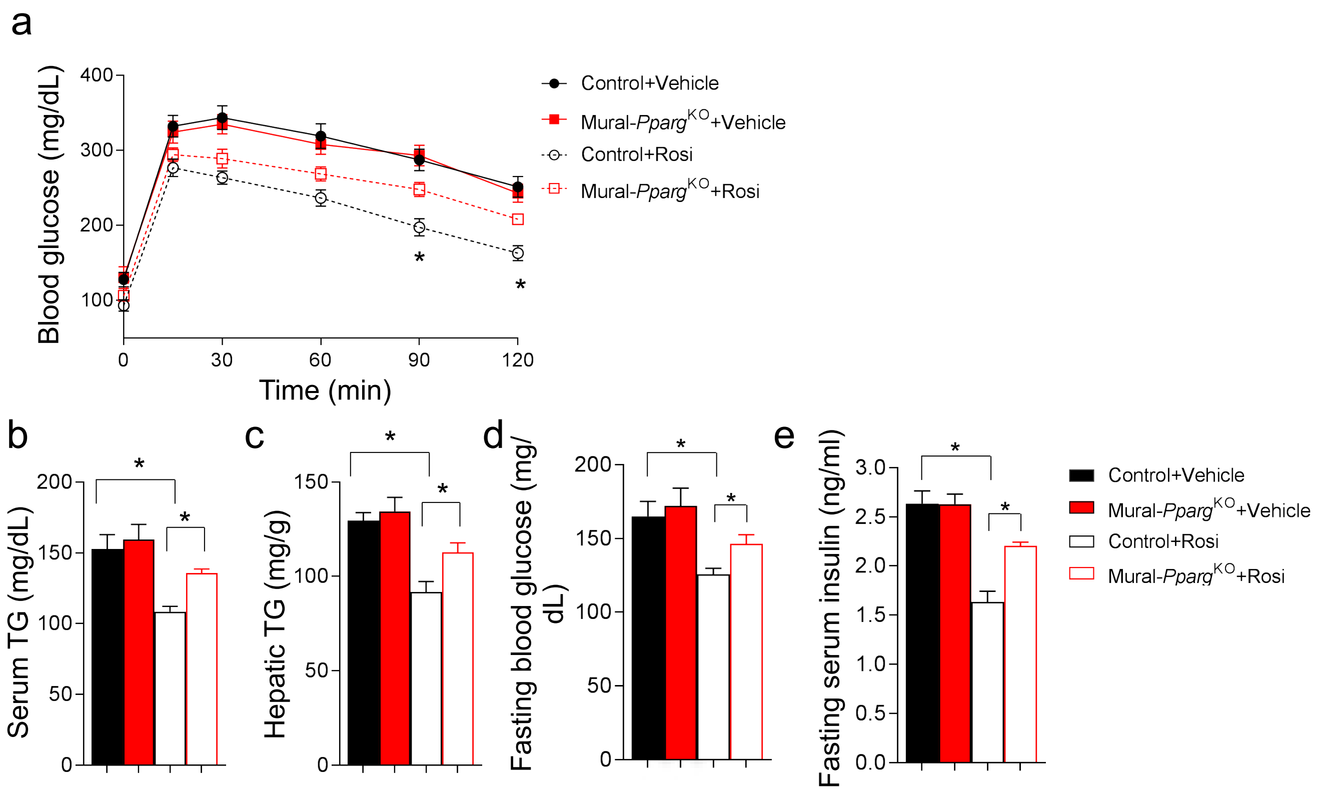


**Supplementary Figure 11: Mural cell *Pparg* is required for the full metabolic benefit of rosiglitazone.**

**(a)** Glucose tolerance tests of obese control and Mural-*Pparg*$^{KO}$ mice after 4 weeks of vehicle or rosiglitazone treatment. Two-way ANOVA, *p< 0.05; Control+Vehicle, n=8; Mural-*Pparg*$^{KO}$ +Vehicle, n=8; Control+Rosi, n=8; Mural-*Pparg*$^{KO}$ +Rosi, n=9. Data points represent mean $\pm$ s.e.m.

**(b)** Serum and **(c)** hepatic triglycerides levels in obese control and Mural-*Pparg*$^{KO}$ mice after 4 weeks of vehicle or rosiglitazone treatment. Two-way ANOVA, *p< 0.05; Control+Vehicle, n=8; Mural-*Pparg*$^{KO}$ +Vehicle, n=8; Control+Rosi, n=8; Mural-*Pparg*$^{KO}$ +Rosi, n=9. Bars represent mean $\pm$ s.e.m.

**(d)** 6-hour fasting blood glucose levels of obese control and Mural-*Pparg*$^{KO}$ mice treated with vehicle or rosiglitazone. Two-way ANOVA, *p< 0.05. Control+Vehicle, n=8; Mural-*Pparg*$^{KO}$ +Vehicle, n=9; Control+Rosi, n=11; Mural-*Pparg*$^{KO}$ +Rosi, n=19. Bars represent mean $\pm$ s.e.m.

**(e)** 6-hour fasting serum insulin levels of control and Mural-*Pparg*$^{KO}$ mice treated with vehicle or rosiglitazone. Two-way ANOVA, *p< 0.05. Control+Vehicle, n=8; Mural-*Pparg*$^{KO}$ +Vehicle, n=8; Control+Rosi, n=8; Mural-*Pparg*$^{KO}$ +Rosi, n=9. Bars represent mean $\pm$ s.e.m.

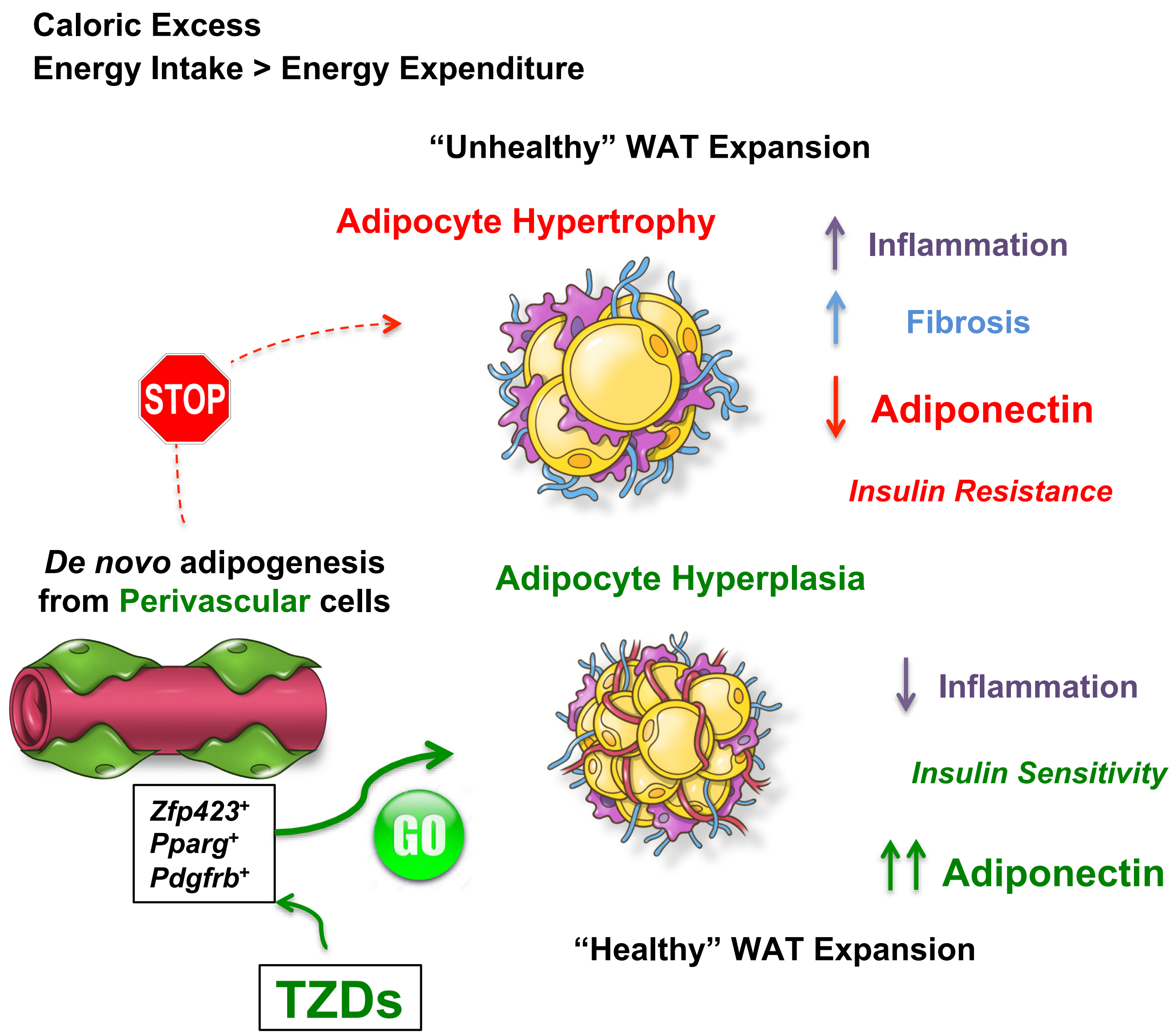


**Supplementary Figure 12: Proposed Model.**

We propose that the adipogenic capacity of Pdgfrβ+ mural cells determines visceral WAT health and serum adiponectin levels in obesity. Committed mural (Pdgfrβ$^+$) adipocyte precursors reside in adipose depots of adult mice and can be defined by the expression of *Pparg* and its upstream regulator, *Zfp423*. In the setting of caloric excess (high-fat diet), Pdgfrβ+ precursors undergo white adipocyte differentiation in visceral WAT depots. The inability of these cells to undergo adipogenesis (e.g. loss of *Pparg* expression) leads to a pathological expansion of WAT, characterized predominantly by macrophage infiltration, tissue fibrosis, reduced tissue insulin sensitivity, and loss of adiponectin production. Driving adipocyte differentiation from Pdgfrβ$^+$ mural cells (e.g. *Pparg* overexpression or activation by TZDs) leads to visceral adipocyte hyperplasia at the expense of adipocyte hypertrophy. This healthy visceral WAT remodeling is associated with less inflammation, maintenance of tissue insulin sensitivity, and a preservation of adiponectin levels. Graphic illustrations of adipose tissue were reproduced from *Molecular and Cellular Endocrinology*, Vol 445: 95-108, 2017, with permission from Elsevier.

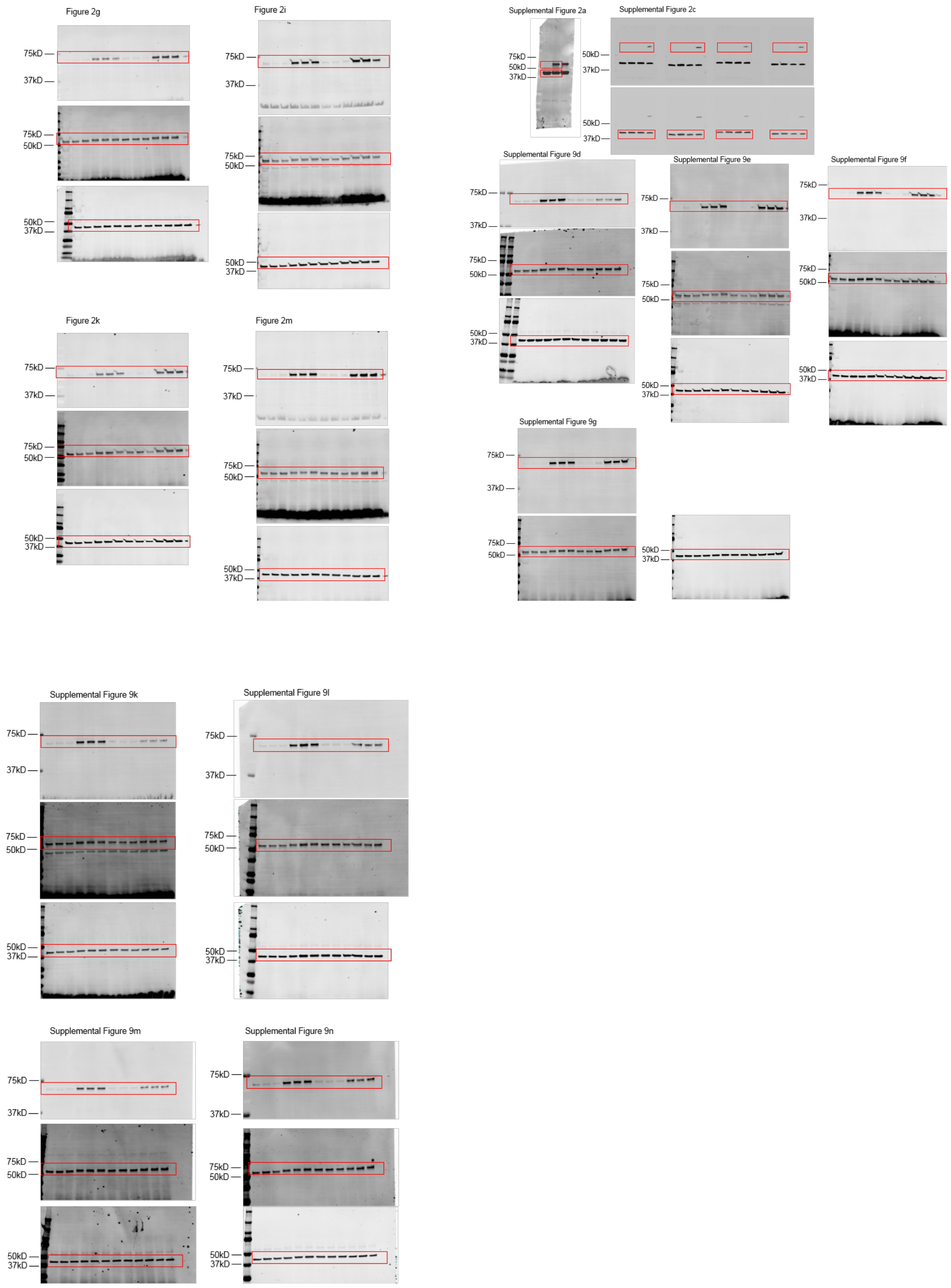


**Supplementary Figure 13: Full-length western blot images**

**Supplementary Table 1: Sequences of qPCR primers used in this study**

| Gene | Forward 5'-3' | Reverse 5'-3' |
|---|---|---|
| *Pparg2* | GCATGGTGCCTTCGCTGA | TGGCATCTCTGTGTCAACCATG |
| *Cebpa* | TGGCCTGGAGACGCAATGA | CGCAGAGATTGTGCGTCTTT |
| *Adiponectin* | AGATGGCACTCCTGGAGAGAA | TTCTCCAGGCTCTCCTTTCCT |
| *Fabp4* | GATGAAATCACCGCAGACGAC | ATTCCACCACCAGCTTGTCAC |
| *Adipsin* | CTACATGGCTTCCGTGCAAGT | AGTCGTCATCCGTCACTCCAT |
| *Ucp1* | TCTCAGCCGGCTTAATGACTG | GGCTTGCATTCTGACCTTCAC |
| *Fgf21* | CTGCTGGGGGTCTACCAAG | CTGCGCCTACCACTGTTCC |
| *Elovl3* | GTGTGCTTTGCCATCTACACG | CTCCCAGTTCAACAACCTTGC |
| *Cidea* | TCCTATGCTGCACAGATGACG | TGCTCTTCTGTATCGCCCAGT |
| *Cox8b* | TGCTGGAACCATGAAGCCAAC | AGCCAGCCAAAACTCCCACTT |
| *Col1a1* | AGATGATGGGGAAGCTGGCAA | AAGCCTCGGTGTCCCTTCATT |
| *Col3a1* | ATTCTGCCACCCCGAACTCAA | ACAGTCATGGGGCTGGCATTT |
| *Col5a1* | TGTCATGTTTGGCTCCCGGAT | AGTCATAGGCAGCTCGGTTGT |
| *Acta2* | TGACGCTGAAGTATCCGATAGA | GTACGTCCAGAGGCATAGAGG |
| *Tnfa* | CCTGTAGCCCACGTCGTAG | GGGAGTAGACAAGGTACAACCC |
| *IL6* | TAGTCCTTCCTACCCCAATTTCC | TTGGTCCTTAGCCACTCCTTC |
| *F4/80* | TGACTCACCTTGTGGTCCTAA | CTTCCCAGAATCCAGTCTTTCC |
| *Cd115* | TGTCATCGAGCCTAGTGGC | CGGGAGATTCAGGGTCCAAG |
| *Cd11b* | GGCTCCGGTAGCATCAACAA | ATCTTGGGCTAGGGTTTCTCT |
| *iNOS* | ACATCGACCCGTCCACAGTAT | CAGAGGGGTAGGCTTGTCTC |
| *Cd11c* | GTGCTGAGTTCGGACACAGT | AGAGGCCACCTATTTGGTTAGT |
| *Mrc1* | CTCTGTTCAGCTATTGGACGC | CGGAATTTCTGGGATTCAGCTTC |
| *Ym1* | CAGGTCTGGCAATTCTTCTGAA | GTCTTGCTCATGTGTGTAAGTGA |
| *Arg1* | CTCCAAGCCAAAGTCCTTAGAG | AGGAGCTGTCATTAGGGACATC |
| *Cd31* | ACGCTGGTGCTCTATGCAAG | TCAGTTGCTGCCCATTCATCA |
| *VE-Cad* | CCAACGTGAACCGCCAGAA | GTGTTAGCATCGACCCCGAA |
| *Pdgfrb* | CAAGAAGCGGCCATGAATCAG | CGGCCCTAGTGAGTTGTTGT |
| *Rps18* | CATGCAAACCCACGACAGTA | CCTCACGCAGCTTGTTGTCTA |
